# The Association of Intra-Operative Steroid Administration on Hearing Threshold and Speech Perception Outcomes in Adult Cochlear Implant Recipients: A Retrospective Cohort Study

**DOI:** 10.3390/audiolres16050142

**Published:** 2026-09-23

**Authors:** Sofia Anastasiadou, Emma Turner, Paris Bruno, Mihaela Gorea, Majed Yousif, Linnea Cheung

**Affiliations:** 1 Ear Nose and Throat, Royal Infirmary, Bristol BS2 8HW, UK; 2 Bristol Medical School, University of Bristol, Bristol BS2 8HW, UK

**Keywords:** cochlear implantation, intra-operative steroids, pure tone audiometry, speech perception, hearing outcomes

## Abstract

**Background:** Intra-operative steroid administration during cochlear implantation is intended to reduce cochlear inflammation and preserve auditory function, but clinical evidence of benefit remains inconsistent. This study evaluated the association between intra-operative steroid use and post-operative pure tone audiometry (PTA) thresholds and speech perception outcomes in adult cochlear implant recipients. **Methods:** A retrospective cohort study was conducted including 370 adults with complete PTA data and 332 with interpretable speech perception scores. Patients were stratified by intra-operative steroid use (PTA: n = 112; speech: n = 96) versus no steroid use (PTA: n = 258; speech: n = 236). Post-operative PTA represented unaided residual acoustic hearing, measured without the cochlear implant at 500, 1000, 2000, 4000 and 8000 Hz. Speech perception was assessed using BKB and AB tests in quiet, with the CI alone, at three months after CI activation, using the same testing approach for all patients. Between-group comparisons were performed using parametric and non-parametric tests. Analysis of covariance (ANCOVA) was used to adjust for baseline performance. Sensitivity analyses and subgroup analyses stratified by pre-operative speech scores were undertaken. **Results:** Residual acoustic PTA improved substantially in both groups, with no significant between-group difference (steroid: 27.1 dB vs. no-steroid: 30.4 dB; *p* = 0.107). ANCOVA confirmed no independent association between steroid use and PTA outcome (β = −0.14 dB, 95% CI −1.80 to +1.52; *p* = 0.869). In contrast, the steroid group demonstrated lower post-operative speech perception (64.5% vs. 71.3%; *p* = 0.048) and smaller improvement from baseline (36.6 vs. 47.1 percentage points; *p* = 0.004). After adjustment, steroid use was associated with an 8.4 percentage point lower speech score (95% CI 2.0 to 14.8; *p* = 0.010). This finding was consistent across multiple sensitivity analyses. Subgroup analyses did not show statistically significant differences in most baseline strata, although a significant association was observed in patients with higher pre-operative speech scores. Given the observational design, this association may reflect unmeasured confounding, including surgical selection factors. **Conclusions:** Intra-operative steroid use was not associated with improved PTA outcomes in this cohort. An observed association with lower speech perception outcomes is likely influenced by residual confounding and should be interpreted with caution. Prospective, standardised studies are required to determine the role of intra-operative steroids in cochlear implantation.

## 1. Introduction

Cochlear implantation (CI) is the established standard of care for adults with severe-to-profound sensorineural hearing loss who derive limited benefit from conventional amplification. Over the past two decades, expanding candidacy criteria have led to an increasing proportion of implanted patients with residual low-frequency hearing, placing greater emphasis on surgical strategies that protect cochlear microstructure and preserve residual acoustic function.

The surgical trauma inherent to cochlear implantation—including drilling, perilymph displacement, and electrode insertion—is known to provoke an acute inflammatory cascade within the cochlea. This response is characterised by increased endocochlear fluid cytokine concentrations, fibrous tissue deposition around the electrode array, and, in some patients, progressive loss of residual hearing in the weeks to months following surgery [1,2]. Intra-operative steroid administration, typically via systemic intravenous delivery or local intratympanic or intracochlear instillation, has been proposed as a means of suppressing this inflammatory response, thereby protecting cochlear hair cells and spiral ganglion neurons [3].

Despite the biological rationale for this intervention, clinical evidence for its efficacy has been mixed. Several studies have reported improvements in residual hearing preservation with steroid use, particularly at low frequencies, while others have found no significant difference in post-operative audiological outcomes compared to controls [4,5,6]. Heterogeneity in steroid type, route of administration, dosing, timing, and the audiological outcome measures used has made cross-study comparisons difficult [7]. Notably, most existing studies have focused exclusively on hearing threshold measurements and have not systematically evaluated the impact of intra-operative steroids on speech perception—the functional outcome of greatest importance to patients.

This study aimed to address this gap by evaluating the effect of intra-operative steroid administration on two complementary outcomes in a single adult CI cohort: post-operative pure tone audiometry (PTA) hearing thresholds and speech perception scores. The specific objectives were to determine whether steroid use was associated with significantly different PTA improvement, post-operative speech perception, and the proportion of patients reaching clinically meaningful outcome thresholds, and to assess whether any observed differences persisted after adjustment for baseline auditory performance. Given the retrospective, non-standardised allocation of steroids, the study was designed as an observational analysis rather than a test of causal treatment efficacy.

## 2. Methods

### 2.1. Study Design and Patient Selection

A retrospective cohort study was conducted at a tertiary adult cochlear implant centre. All adult patients (≥18 years) who underwent unilateral or bilateral cochlear implantation and had documented pre- and post-operative audiological assessments were eligible for inclusion. Patients were identified from the programme’s clinical database and stratified according to whether intra-operative topical dexamethasone was administered at the time of surgery. Dexamethasone 4 mg/mL was used topically, applied to the round window niche/middle ear at the surgeon’s discretion; systemic intravenous steroids were not part of the standard institutional protocol during the study period and were not separately recorded, so this analysis addresses topical/intratympanic administration only and cannot comment on the additive or independent effect of intravenous steroids used at many other centres. The exact duration of topical application, the specific surgical approach (e.g., round window versus cochleostomy), and other technical variables known to influence audiological outcome were not systematically documented in the clinical database and could therefore not be included as covariates; this is acknowledged as a limitation below. The decision to administer steroids was made according to surgeon preference and was not subject to a standardised allocation protocol during the study period, reflecting routine clinical practice. Four surgeons performed cochlear implants regularly, and all patients were included to minimise operator-related selection of the study cohort. Three CI manufacturers were represented (Cochlear, Advanced Bionics and MED-EL), although electrode-array-level data and postoperative electrode position were not incorporated into the present analysis.

Two analytical cohorts were defined. The primary cohort comprised all patients with complete pre- and post-operative residual acoustic PTA data (n = 370). The secondary cohort comprised patients with valid, interpretable pre- and post-operative speech perception scores (n = 332); 38 patients were excluded from speech analysis due to non-numeric or uninterpretable entries (e.g., “NO REPORT”, “NT”) or entries in formats that could not be reliably converted to a single percentage score. Where speech scores were recorded in a dual-condition format, the mean of the two values was used. Post-operative speech perception was assessed three months after CI activation, providing a common predefined clinical timepoint across the cohort.

### 2.2. Audiological Outcome Measures

The primary PTA outcome was change in residual acoustic hearing, defined as the arithmetic difference between pre-operative and post-operative PTA thresholds in dB HL (positive values indicating improvement). Post-operative PTA was measured without the CI, using ear-specific (not sound-field) unaided air-conduction thresholds at 500, 1000, 2000, 4000 and 8000 Hz, obtained in a sound-treated booth as part of routine clinical audiological review; centralised calibration records for the audiometric equipment across the full study period were not available for this retrospective analysis, and this is acknowledged as a limitation. Secondary PTA outcomes included absolute post-operative residual-hearing PTA and the proportion of patients achieving a clinically meaningful improvement of ≥10 dB. PTA was therefore treated as a measure of residual acoustic hearing rather than a CI fitting or electrical stimulation measure, and is explicitly distinguished in this study from speech perception outcomes (below): the two are complementary but non-interchangeable measures, and neither is used here as a proxy for the other.

The primary speech perception outcome was the post-operative CI-alone open-set speech score expressed as a percentage. Speech perception was assessed using BKB and AB tests in quiet at three months after CI activation; both are open-set, sentence/word-based tests scored as percentage correct in the same clinical pathway, and scores were pooled as a single percentage-correct outcome without a formal cross-test equivalence adjustment. The same speech-testing approach was used for all patients, and pooling the two tests, while reflecting real-world clinical practice, may introduce some additional outcome variability, which is acknowledged as a limitation. Secondary outcomes included absolute speech improvement (post-operative minus pre-operative score) and the proportions of patients achieving post-operative speech scores of ≥50% and ≥80%, the latter representing a commonly cited threshold for functional open-set speech understanding in quiet.

### 2.3. Statistical Analysis

Continuous variables are reported as mean ± standard deviation (SD) unless otherwise stated. Between-group comparisons were performed using the independent-samples *t*-test for normally distributed outcomes and the Mann–Whitney U test as a non-parametric alternative, given evidence of non-normality in the speech improvement distributions (Shapiro–Wilk test). The *t*-test was retained as the principal parametric comparison because of the large sample size and its robustness to moderate deviations from normality. Categorical outcomes were compared using the chi-squared test.

Analysis of covariance (ANCOVA) was the primary inferential model for both PTA and speech outcomes, with intra-operative steroid use as the factor of interest and pre-operative baseline performance (PTA or speech score, respectively) as the covariate. The ANCOVA model partitioned between-group variance from that attributable to baseline performance, allowing estimation of the adjusted group difference. The regression coefficient (β) from the ANCOVA model represents the adjusted group difference in outcome, with 95% confidence intervals and an associated F-statistic. Effect size was quantified using Cohen’s d for continuous outcomes, with values of 0.2, 0.5, and 0.8 representing small, medium, and large effects, respectively. A two-sided *p*-value of <0.05 was considered statistically significant. All analyses were performed using Python 1.18.1 version (SciPy and NumPy libraries). Because steroid administration was determined by surgeon preference rather than randomisation, ANCOVA was considered an adjustment for baseline performance only and not a method capable of eliminating unmeasured confounding.

Regarding the speech perception outcome, further analysis was conducted to assess whether the primary association was robust to influential observations and baseline heterogeneity. Five pre-specified sensitivity analyses were performed: IQR-based exclusion, trimming of the bottom and top 2.5% of improvement scores, trimming of the bottom and top 5%, exclusion of patients with a post-operative score of zero, and Winsorisation at the 2.5th and 97.5th percentiles. Subgroup analyses were conducted by stratifying patients into four pre-operative baseline speech bands (0%, 1–20%, 21–50%, and >50%) and by tertile split, to determine whether any observed association varied according to pre-operative performance. Within the high-baseline tertile (>32%), improvement was additionally expressed as a proportion of available ceiling capacity (defined as 100 minus the pre-operative score) to assess whether the observed difference persisted after accounting for differential headroom. These analyses were considered exploratory and no formal correction for multiple comparisons was applied.

## 3. Results

### 3.1. Patient Demographics and Baseline Characteristics

A total of 370 adult patients were included in the PTA analysis. The mean age at assessment was 69.4 years (SD ± 17.0), and the mean age at implantation was 62.7 years (SD ± 16.6; range 16–98 years) (Table 1). The cohort was predominantly of White British ethnicity (96.5%). Of the 370 patients, 184 (49.7%) were male and 184 were female (one record contained an erroneous gender entry and was excluded from gender-specific analyses). The majority underwent unilateral implantation (steroid group: 60 left-sided, 50 right-sided, 5 bilateral; no-steroid group: 117 left-sided, 134 right-sided, 6 bilateral). Seventy percent of patients in the steroid group had a pre-operative MRI compared with 77.9% in the no-steroid group.

Intra-operative topical dexamethasone was administered in 112 patients (30.3%). Baseline demographic and clinical characteristics were comparable between groups. Mean age at implantation was 62.4 years (SD ± 16.2) in the steroid group and 62.8 years (SD ± 16.9) in the no-steroid group. Male sex accounted for 54.1% of the steroid group and 48.2% of the no-steroid group. Pre-operative PTA thresholds were 57.9 dB (SD ± 16.9) in the steroid group and 61.4 dB (SD ± 18.2) in the no-steroid group; this difference did not reach statistical significance (*t*-test: *p* = 0.092). For the speech cohort (n = 332), baseline speech perception scores were 27.9% (SD ± 25.8) in the steroid group and 24.2% (SD ± 23.1) in the no-steroid group (*p* = 0.200) (Table 2). Cochlear, Advanced Bionics and MED-EL devices were represented in the cohort; array-level distributions were not included in the present analysis.

### 3.2. PTA Outcomes

Both groups demonstrated substantial improvements in residual acoustic PTA following cochlear implantation. In the steroid group, mean PTA improved from 57.9 dB to 30.9 dB (improvement of 27.1 dB, SD ± 18.6). In the no-steroid group, mean PTA improved from 61.4 dB to 30.9 dB (improvement of 30.4 dB, SD ± 18.4). Because postoperative PTA was measured without the CI, these values represent residual acoustic hearing rather than CI programming or electrical stimulation thresholds. Post-operative residual-hearing PTA was virtually identical between groups (30.9 dB in both; *p* = 0.965) (Table 3).

The difference in residual-hearing PTA improvement between groups was not statistically significant (*t*-test: t = −1.61, *p* = 0.107; Mann–Whitney U: *p* = 0.200). ANCOVA adjusting for pre-operative PTA confirmed the absence of an independent steroid association (β = −0.14 dB, 95% CI −1.80 to +1.52, F = 0.03, *p* = 0.869) (Figure 1). A clinically meaningful hearing improvement of ≥10 dB was achieved by 91.1% of patients in the steroid group and 93.0% in the no-steroid group; this difference was not significant (χ^2^ = 0.19, *p* = 0.661). Cohen’s d for PTA improvement was −0.18, indicating a negligible effect size. Pre-operative PTA was the only significant predictor of outcome in the ANCOVA model, with greater pre-operative hearing loss associated with larger post-operative improvements.

### 3.3. Speech Perception Outcomes

Both groups showed substantial gains in CI-alone speech perception following cochlear implantation. Speech perception was assessed using BKB and AB tests in quiet at three months after activation, under the same testing approach for all patients. Within-group paired analyses confirmed highly significant improvements in both the steroid group (pre-operative: 27.9%, post-operative: 64.5%; t = −11.32, *p* < 0.001) and the no-steroid group (pre-operative: 24.2%, post-operative: 71.3%; t = −24.64, *p* < 0.001). However, the magnitude of improvement differed between groups (Table 4). Mean post-operative speech perception was 64.5% (SD ± 29.8; median 73%) in the steroid group compared with 71.3% (SD ± 27.9; median 80%) in the no-steroid group (*t*-test: t = −1.99, *p* = 0.048; Mann–Whitney U: *p* = 0.033). Mean speech improvement from baseline was 36.6 percentage points (SD ± 31.6) in the steroid group versus 47.1 percentage points (SD ± 29.4) in the no-steroid group (*t*-test: t = −2.91, *p* = 0.004; Mann–Whitney U: *p* = 0.006). ANCOVA adjusting for baseline speech score estimated an adjusted group difference of 8.4 percentage points (95% CI 2.0 to 14.8, F = 6.74, *p* = 0.010), with a small-to-moderate effect size (Cohen’s d = −0.35). A significantly lower proportion of patients in the steroid group achieved a post-operative score of ≥80% (37.5% vs. 52.5%; χ^2^ = 5.60, *p* = 0.018). Because all speech testing was performed at three months after activation, timing was standardised within the cohort; however, individual variation in early CI adaptation cannot be excluded.

Additional analyses were undertaken to assess whether the observed association was robust or concentrated in particular baseline groups. Subgroup analysis by baseline speech score was used to explore whether the difference varied across levels of pre-operative function, while sensitivity analyses assessed whether extreme observations disproportionately influenced the result. Because baseline scores can influence the amount of possible improvement, a regression-to-the-mean and ceiling-capacity perspective was also considered. These analyses were exploratory and were intended to qualify, rather than establish, a causal interpretation of the primary association.

All five outlier sensitivity analyses retained a statistically significant adjusted association, with ANCOVA-adjusted differences ranging from −6.6 to −8.0 percentage points and *p*-values between 0.009 and 0.036. Only four improvement-score outliers were identified by the IQR method, three of which belonged to the steroid group; their removal reduced rather than amplified the group difference. These analyses therefore demonstrate directional robustness of the observed association, but do not exclude systematic confounding.

Subgroup analysis by pre-operative baseline speech band revealed that most baseline subgroups did not reach statistical significance individually, although a significant difference was observed in patients with higher pre-operative speech scores (>32%) (Figure 2). No formal correction for multiple comparisons was applied; findings should therefore be interpreted as exploratory. The difference was absent in patients with a floor baseline of 0% (steroid improvement: 48.4 vs. no-steroid: 53.5 percentage points; *p* = 0.550), non-significant in those with baseline scores of 1–20% (48.9 vs. 60.5; *p* = 0.064) and 21–50% (35.1 vs. 44.0; *p* = 0.079), and non-significant in those with baseline scores above 50% (10.5 vs. 18.5; *p* = 0.210). Tertile analysis similarly showed non-significant differences in the low (≤8%; *p* = 0.369) and mid (8–32%; *p* = 0.182) tertiles. The only subgroup reaching significance was patients with pre-operative speech above 32% (steroid: 16.7 vs. 30.0 percentage points improvement; *p* = 0.008, Cohen’s d = −0.55). Baseline scores within this subgroup were comparable between groups (steroid: 56.9% vs. no-steroid: 53.0%; *p* = 0.195). When improvement was expressed as a proportion of available ceiling capacity, steroid patients realised 39.6% of their remaining potential compared with 56.9% in the no-steroid group (Mann–Whitney U: *p* = 0.016). These findings suggest a potentially important subgroup pattern, but the analysis remains exploratory and cannot establish whether steroid exposure itself caused the difference [8].

## 4. Discussion

This retrospective cohort study of 370 adult cochlear implant recipients provides two principal findings. First, intra-operative steroid administration had no significant difference on post-operative PTA hearing thresholds, with no difference detected in hearing improvement, absolute post-operative threshold, or the proportion reaching a clinically meaningful threshold, and with a negligible effect size (Cohen’s d = −0.18). Second, and unexpectedly, intra-operative steroids were associated with significantly poorer post-operative speech perception outcomes, with a mean adjusted deficit of 8.4 percentage points that persisted after controlling for baseline speech performance. This discordance between PTA and speech outcomes is the most striking aspect of these data and warrants careful consideration.

The absence of a steroid benefit on residual acoustic hearing is broadly consistent with several prior studies that have failed to demonstrate a significant advantage of steroid administration on post-operative audiometric outcomes in adult CI populations [8]. Possible explanations include the fact that electrode insertion trauma and inflammatory cochlear fibrosis may be sufficiently variable in their severity that a uniform pharmacological intervention of modest potency does not produce a detectable population-level effect. It is also possible that the timing and route of steroid delivery is a critical determinant of efficacy. The present study used topical dexamethasone 4 mg/mL according to surgeon preference, rather than a randomised protocol, so the findings cannot determine whether another dose, timing or route would have produced a different result. Importantly, the postoperative PTA used here was measured without the CI and therefore was not a fitting-dependent electrical threshold. It directly assessed residual acoustic hearing across 500–8000 Hz. This distinction addresses an important methodological issue in interpreting postoperative PTA after implantation: the present PTA outcome should not be described as a measure of perceived loudness of electrical stimulation or CI mapping performance.

With that caveat, the ANCOVA finding (β = −0.14 dB, 95% CI −1.80 to +1.52) is notable for the narrowness of its confidence interval: even the upper bound represents a benefit of only 1.5 dB, a value that is unlikely to be clinically meaningful. The data therefore provide reasonable grounds for concluding that, in this cohort, any effect of intra-operative steroids on PTA was trivially small.

However, multiple studies have tried to investigate effects of hearing preservation techniques specifically for lower frequencies adopting different techniques and methods of steroid administration [9,10,11]. Currently there is not any robust evidence to support a reliable audiometric benefit from perioperative steroid administration in adult cochlear implant recipients. A recent systematic review of the literature including a total number of 659 implanted patients demonstrated mild superiority of short lived hearing and vestibular outcomes with intra-operative topical steroid administration [12]. Although several studies have reported improved hearing preservation rates with steroid regimens—particularly with topical or intratympanic delivery and with multi-day perioperative oral courses—single intra-operative doses have generally shown weaker and less consistent effects [13]. A critical determinant of steroid efficacy identified in the animal and clinical literature is also the timing and route of delivery, with pre-operative topical round window application appearing to confer greater benefit than systemic intra-operative dosing [14]. Randomised controlled trial data have similarly failed to support the systemic administration practice: at least one double-blind placebo-controlled trial found that high-dose intravenous methylprednisolone at induction conferred no advantage in hearing preservation at any follow-up timepoint [15]. The insertion speed of the electrodes has also been investigated with variable results [16] as well as the placement of the cochleostomy in relation to the round window [14]. In terms of steroid route and efficacy in our study, the present ANCOVA-adjusted estimate of just 1.5 dB is unlikely to carry any clinical significance and the consistency of this finding across unadjusted comparisons, non-parametric testing, and the covariance-adjusted model strengthens confidence in the conclusion. These findings do not establish that device characteristics explain the present steroid association, but they reinforce the need to record manufacturer, array design, insertion depth and intracochlear position in future prospective comparisons. when evaluating postoperative speech perception as the primary functional outcome.

The finding of lower speech perception outcomes in the steroid group is less easily attributed to direct pharmacological harm and requires cautious interpretation. The most important methodological caveat is that this is a non-randomised observational study in which steroid use reflected surgical judgement rather than a predetermined protocol. Patients receiving steroids may have been perceived intra-operatively as being at higher risk—due to factors such as greater cochlear ossification, a more traumatic electrode insertion, or the presence of relevant comorbidities—and it is these underlying characteristics rather than the steroids themselves that may be responsible for the worse speech outcomes. This selection bias, which is inherent to any retrospective analysis of an unblinded clinical decision, cannot be fully adjusted for in the statistical model. The ANCOVA controlled for baseline speech performance, confirming that the difference was not attributable to worse pre-operative function in the steroid group, but residual confounding by unmeasured surgical variables remains a significant limitation.

The magnitude of the speech difference—approximately 8 percentage points after adjustment, with a Cohen’s d of −0.35—is clinically modest but not trivial. At the threshold of 80% correct open-set speech in quiet, the proportion achieving this benchmark was 37.5% in the steroid group versus 52.5% in the no-steroid group, a 15 percentage point absolute difference. For individual patients, the difference between a score of 65% and 80% represents a meaningful real-world impact on communication ability in daily life. Whether this difference reflects a causal steroid effect, surgical case complexity, or an interaction between the two cannot be determined from the present data.

The dissociation between PTA and speech outcomes observed here is of potential scientific interest. It is conceivable that steroids exert differential effects on the cochlear and neural substrates underlying threshold detection versus suprathreshold speech processing. PTA thresholds are largely determined by outer hair cell function and cochlear mechanics, while speech perception in implant users depends primarily on the integrity and excitability of spiral ganglion neurons, the fidelity of the electrode-neural interface, and central auditory processing. If steroids alter any of these parameters—for example, by transiently modifying endocochlear fluid homeostasis or affecting neuronal excitability during the critical early post-operative period—this could theoretically affect speech outcomes without a corresponding change in detection thresholds. This hypothesis is speculative and would require controlled prospective investigation to evaluate.

There is however room for further analysis of the results to evaluate the difference and investigate the significantly worse speech perception outcomes in the steroid group. Additional sensitivity and subgroup analyses reported here allows a considerably more nuanced interpretation than the primary result alone would suggest. Across all outlier-removal strategies—IQR exclusion, tail trimming at the 2.5th and 97.5th percentiles, exclusion of post-CI scores of zero, and Winsorisation—the finding remained statistically significant with ANCOVA-adjusted deficits ranging from −6.6 to −8.0 percentage points and *p*-values consistently below 0.04. Critically, only four statistical outliers were identified by the IQR method, three of which belonged to the steroid group; their removal therefore reduced rather than inflated the apparent group difference, confirming that the finding is not an artefact of extreme values. However, the subgroup analysis stratified by pre-operative baseline speech score reveals an important pattern that substantially qualifies this conclusion. When the cohort was divided into four baseline bands, most baseline subgroups did not reach statistical significance individually, although a significant difference was observed in patients with higher pre-operative speech scores (>32%). The difference was entirely absent in patients with a floor baseline score of 0% (*p* = 0.550), non-significant in those with baseline scores of 1–20% (*p* = 0.064) and 21–50% (*p* = 0.079), and non-significant in those with higher pre-operative speech scores above 50% (*p* = 0.210). Tertile analysis confirmed that the only subgroup in which a significant difference emerged was patients with pre-operative speech above 32% (*p* = 0.008, Cohen’s d = −0.55), while both lower tertiles were non-significant. The overall significance in the pooled cohort therefore arises from the accumulation of a consistent directional trend—the steroid group performed worse in every subgroup—rather than from a dominant effect in any single stratum. The localisation of the effect to the high-baseline subgroup warrants careful interpretation and cannot simply be attributed to a ceiling effect. Within this tertile, baseline speech scores were near-identical between groups (steroid 56.9% vs. no-steroid 53.0%, *p* = 0.195), meaning the groups had comparable headroom for improvement. When improvement was expressed as a proportion of available ceiling capacity—that is, how much of the remaining potential each group realised—steroid patients captured only 39.6% of their available room compared to 56.9% in the no-steroid group, a difference that reached significance on non-parametric testing (Mann–Whitney *p* = 0.016). This suggests that even after accounting for starting position and ceiling constraints, steroid patients in this subgroup under-performed relative to their available potential. The most plausible explanation remains confounding by surgical case characteristics: in routine clinical practice, intra-operative steroids are most likely to be administered in cases where the surgeon perceives greater cochlear trauma or insertion difficulty—and these are precisely the cases where the electrode-neural interface may be compromised in ways that affect suprathreshold speech processing without necessarily altering detection thresholds. This would account for both the PTA/speech dissociation observed across the full cohort and the concentration of the speech deficit in patients who already had functional hearing, where the stakes—and thus the likelihood of a protective intervention—are highest. The present dataset does not contain sufficient intra-operative detail to test this hypothesis directly, and the finding should be treated as hypothesis-generating. Its statistical robustness across all sensitivity analyses means it cannot be dismissed, but the subgroup data, taken together with the ceiling-effect-corrected analysis, provide a coherent and biologically plausible narrative for why confounding rather than pharmacological harm is the more defensible interpretation.

## 5. Limitations

Several limitations of this study should be acknowledged. The retrospective design and absence of steroid standardisation are the most significant. The cohort is ethnically homogeneous (96.5% White British), limiting generalisability to more diverse populations. Finally, follow-up duration and the interval between implantation and post-operative testing were not uniform across the cohort, which may have introduced outcome variability unrelated to steroid use, as speech perception continues to improve for 12–24 months following activation. Key determinants of speech outcomes such as duration of deafness, electrode positioning, and intra-operative difficulty were not monitored for this study. Three CI manufacturers (Cochlear, Advanced Bionics and MED-EL) were represented in this cohort, and neither manufacturer nor device generation was analysed as a covariate; because steroid use was determined by surgeon preference rather than device type, manufacturer-related confounding is considered unlikely to explain the present findings, but cannot be formally excluded and should be addressed in future work that records array design and generation. This study addresses topical/intratympanic steroid administration only; institutional practice during the study period did not include a standardised intravenous steroid protocol, so these findings cannot be extrapolated to centres in which systemic steroids are routinely combined with topical or intracochlear delivery. Statistical assumptions were checked prior to analysis (Shapiro–Wilk testing for normality, with non-parametric confirmation used throughout), and ANCOVA was used as a multivariable adjustment for baseline performance; formal survival analysis was not applicable, as device survival, revision surgery and explantation were outside the scope of this study, which examines audiological outcomes rather than device longevity.

Notwithstanding these limitations, this study represents one of the larger single-centre retrospective analyses to examine both PTA and speech outcomes in relation to intra-operative steroid use in adult CI recipients. The consistency of the speech finding across multiple statistical approaches—unadjusted *t*-test, non-parametric Mann–Whitney U, ANCOVA, and chi-squared threshold analysis—strengthens confidence that the difference is not a statistical artefact. The PTA null finding is equally consistent across approaches. Taken together, the results call into question the assumption that intra-operative steroid administration produces audiological benefit in adult CI surgery, and raise the hypothesis that it may be a marker for more complex implantations with correspondingly less favourable speech trajectories.

## 6. Conclusions

In this retrospective cohort of adult cochlear implant recipients, intra-operative steroid administration was not associated with improved post-operative PTA hearing thresholds. An association between steroid use and lower post-operative speech perception scores was observed; however, this finding is likely influenced by unmeasured confounding inherent to the non-randomised study design, including potential surgical selection bias.

These results do not provide evidence supporting the routine use of intra-operative steroids for improving audiological outcomes in adult cochlear implantation. The observed differences in speech perception should be considered hypothesis-generating. Prospective, randomised studies with standardised steroid protocols, detailed intra-operative data, and longitudinal follow-up are required to clarify whether intra-operative steroids confer benefit, are neutral, or reflect underlying case complexity.

## Figures and Tables

**Figure 1:**
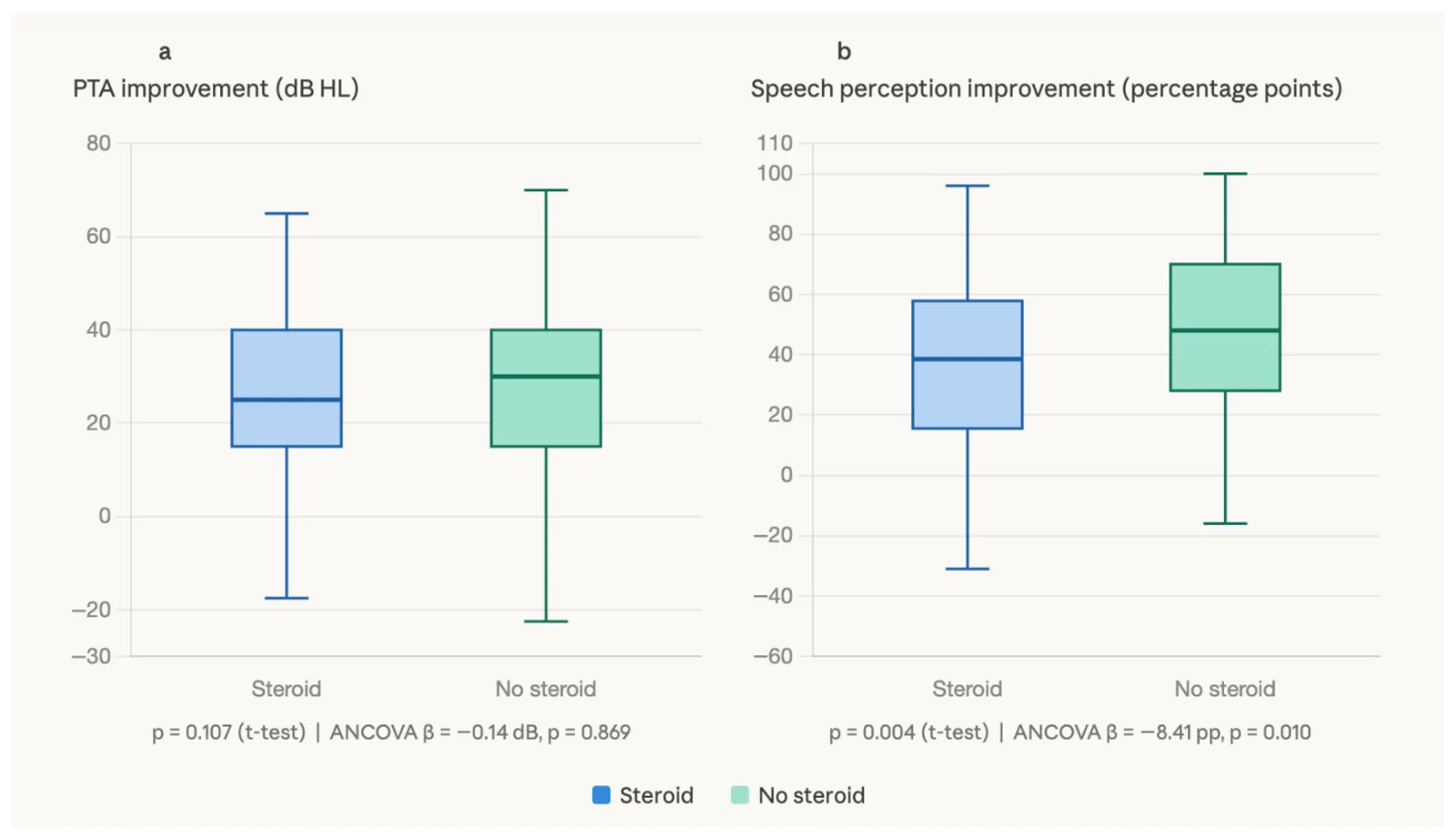
Side-by-side box plots of improvement for PTA (**a**) and speech perception (**b**). The PTA plots are almost identical between groups, visually reinforcing the null finding. The speech plots show a clear downward shift in the steroid group—lower median (38.5 vs. 48 pp), lower Q1, and lower Q3—making the group difference immediately apparent.

**Figure 2:**
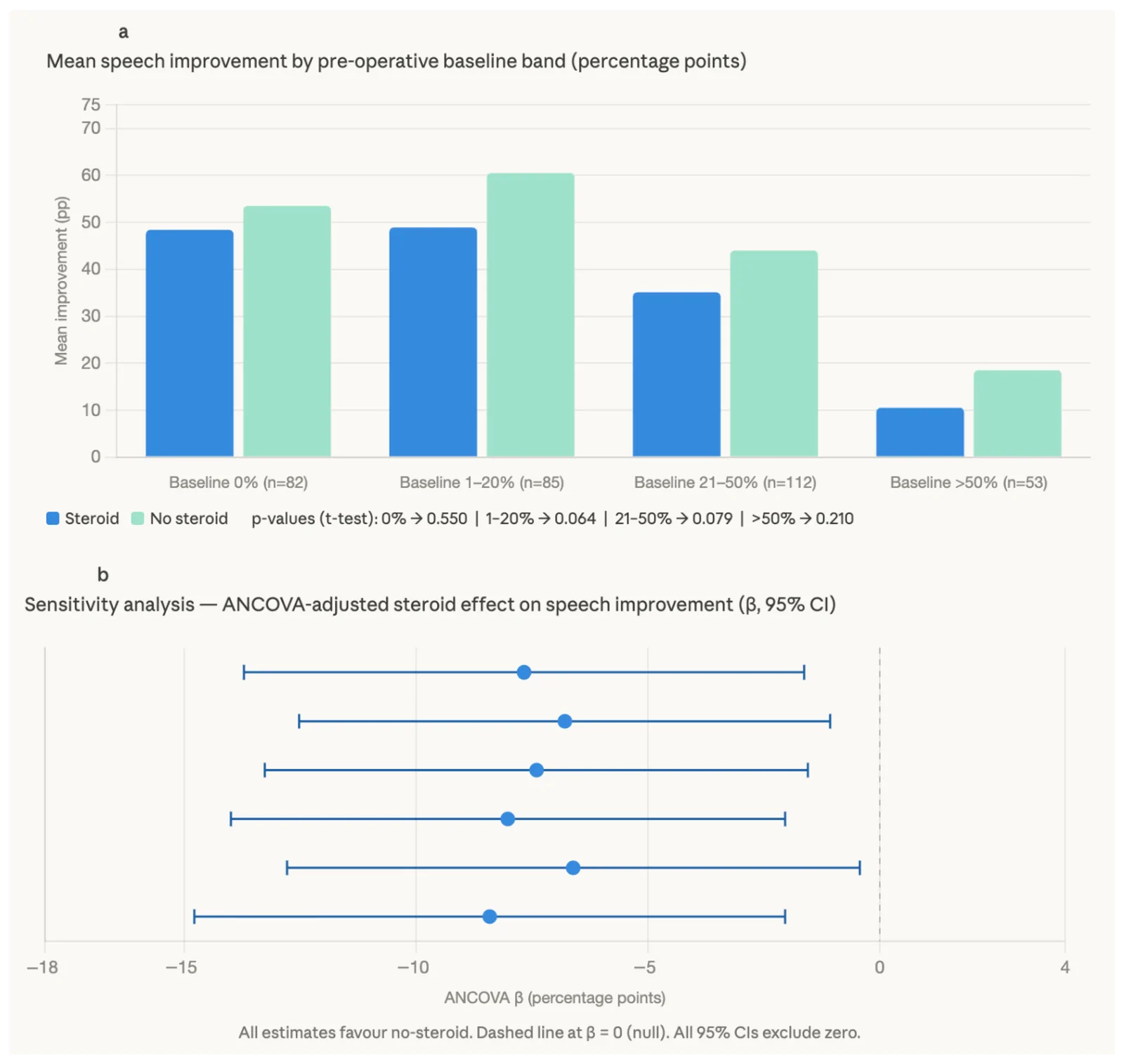
Two panels: (**a**) is a grouped bar chart of mean speech improvement across the four baseline bands, showing the consistent directional gap in every subgroup alongside the *p*-values, making it visually clear that no single band is driving significance on its own. (**b**) is a forest plot of the ANCOVA-adjusted steroid effect (β and 95% CI) across all six sensitivity analyses, demonstrating that every estimate falls in the same region and every confidence interval excludes zero—a compact and rigorous display of robustness.

**Table 1:** Demographics.

Demographics	Steroid (n = 112)	No Steroid (n = 258)	*p*-Value
Age at assessment, years—mean ± SD	67.8 ± 16.9	70.1 ± 17.1	0.251
Age at implantation, years—mean ± SD	62.4 ± 16.2	62.8 ± 16.9	0.854
Male sex—n (%)	60 (54.1%)	124 (48.2%)	0.364
Bilateral implantation—n (%)	5 (4.5%)	6 (2.3%)	0.436
Pre-operative MRI—n (%)	79 (70.5%)	201 (77.9%)	0.166

**Table 2:** Speech perception: subgroup results from further analysis.

Subgroup by Baseline Speech	Values	Values	Values	Values	Values	Values	Values	Values
Baseline 0%	82	48.4 ± 33.9	53.5 ± 35.4	0.550	0.585	—	—	−0.15
Baseline 1–20%	85	48.9 ± 25.0	60.5 ± 24.5	0.064	0.066	−10.93	0.078	−0.47
Baseline 21–50%	112	35.1 ± 30.6	44.0 ± 20.8	0.079	0.199	−7.89	0.099	−0.37
Baseline > 50%	53	10.5 ± 20.5	18.5 ± 22.8	0.210	0.115	−7.11	0.255	−0.36
Low tertile (≤8%)	112	49.7 ± 33.0	56.1 ± 33.8	0.369	0.376	−5.89	0.410	−0.19
Mid tertile (8–32%)	110	47.1 ± 22.2	53.7 ± 23.2	0.182	0.152	−6.63	0.170	−0.29
High tertile (>32%)	110	16.7 ± 27.2	30.0 ± 22.6	0.008	0.007	−10.50	0.021	−0.55

**Table 3:** PTA outcomes.

PTA Outcomes	Values	Values	Values
Pre-operative PTA, dB HL—mean ± SD	57.9 ± 16.9	61.4 ± 18.2	0.092
Post-operative PTA, dB HL—mean ± SD	30.9 ± 7.8	30.9 ± 7.3	0.965
PTA improvement, dB—mean ± SD	27.1 ± 18.6	30.4 ± 18.4	0.107
ANCOVA-adjusted effect (β, 95% CI)	−0.14 (−1.80 to +1.52)	—	0.869
Achieved ≥10 dB improvement—n (%)	102 (91.1%)	240 (93.0%)	0.661

**Table 4:** Speech perception: outlier sensitivity analyses and subgroup results.

Analysis	n	Steroid Improvement	No-Steroid Improvement	*p* (*t*-Test)	*p* (MW)	ANCOVA β	*p* (ANCOVA)	Cohen’s d
Sensitivity analyses								
Full cohort	332	36.6 ± 31.6	47.1 ± 29.4	0.004	0.006	−8.41	0.010	−0.35
IQR outliers removed	328	39.2 ± 28.4	47.6 ± 28.7	0.018	0.015	−6.61	0.036	−0.29
Trim 2.5% tails	315	37.9 ± 25.7	47.7 ± 26.7	0.004	0.004	−8.02	0.009	−0.37
Trim 5% tails	303	38.7 ± 24.4	47.7 ± 25.5	0.005	0.007	−7.40	0.013	−0.36
Exclude post-CI = 0	316	40.1 ± 28.9	49.2 ± 28.2	0.010	0.008	−6.79	0.020	−0.32
Winsorised 2.5/97.5	332	37.7 ± 28.6	47.4 ± 28.4	0.005	0.006	−7.67	0.013	−0.34

## Data Availability

The original contributions presented in this study are included in the article. Further inquiries can be directed to the corresponding author.

## References

[1] Choudhury B., Adunka O.F., Demason C.E., Ahmad F.I., Buchman C.A., Fitzpatrick D.C. (2011). Detection of intracochlear damage with cochlear implantation in a gerbil model of hearing loss. Otol. Neurotol..

[2] De Seta D., Torres R., Russo F.Y., Ferrary E., Kazmitcheff G., Heymann D., Amiaud J., Sterkers O., Bernardeschi D., Nguyen Y. (2017). Damage to inner ear structure during cochlear implantation: Correlation between insertion force and radio-histological findings in temporal bone specimens. Hear. Res..

[3] Rahman M.T., Chari D.A., Ishiyama G., Lopez I., Quesnel A.M., Ishiyama A., Nadol J.B., Hansen M.R. (2022). Cochlear implants: Causes, effects and mitigation strategies for the foreign body response and inflammation. Hear. Res..

[4] Creber N.J., Eastwood H.T., Hampson A.J., O’Leary S.J. (2023). Cochlear implant surgery facilitates intracochlear distribution of perioperative systemic steroids. Acta Otolaryngol..

[5] Lyu A.R., Kim D.H., Lee S.H., Shin D.S., Shin S.A., Park Y.H. (2018). Effects of dexamethasone on intracochlear inflammation and residual hearing after cochleostomy: A comparison of administration routes. PLoS ONE.

[6] Plontke S.K., Götze G., Rahne T., Liebau A. (2017). Intracochlear drug delivery in combination with cochlear implants: Current aspects. HNO.

[7] Chin O.Y., Diaz R.C. (2019). State-of-the-art methods in clinical intracochlear drug delivery. Curr. Opin. Otolaryngol. Head Neck Surg..

[8] Skarżyńska M.B., Skarżyński P.H., Król B., Kozieł M., Osińska K., Gos E., Skarżyński H. (2018). Preservation of Hearing Following Cochlear Implantation Using Different Steroid Therapy Regimens: A Prospective Clinical Study. Med. Sci. Monit..

[9] Sweeney A.D., Carlson M.L., Zuniga M.G., Bennett M.L., Wanna G.B., Haynes D.S., Rivas A. (2015). Impact of Perioperative Oral Steroid Use on Low-frequency Hearing Preservation After Cochlear Implantation. Otol. Neurotol..

[10] Bruce I.A., Felton M., Lockley M., Melling C., Lloyd S.K., Freeman S.R., Green K.M. (2014). Hearing preservation cochlear implantation in adolescents. Otol. Neurotol..

[11] O’Connell B.P., Hunter J.B., Wanna G.B. (2016). The importance of electrode location in cochlear implantation. Laryngoscope Investig. Otolaryngol..

[12] Pimentel de Morais C., Branco P., Pereira A., Castelhano L., Donato M., Correia F., O’Neill A., Santos R., Escada P. (2024). The Role of Steroids in the Preservation of Hearing and Vestibular Function in Cochlear Implantation. Laryngoscope.

[13] Kuthubutheen J., Coates H., Rowsell C., Nedzelski J., Chen J.M., Lin V. (2015). The role of extended preoperative steroids in hearing preservation cochlear implantation. Hear. Res..

[14] Maria P.L.S., Gluth M.B., Yuan Y., Atlas M.D., Blevins N.H. (2014). Hearing preservation surgery for cochlear implantation: A meta-analysis. Otol. Neurotol..

[15] O’Leary S.J., Choi J., Brady K., Matthews S., Ozdowska K.B., Payne M., McLean T., Rousset A., Lo J., Creber N. (2021). Systemic methylprednisolone for hearing preservation during cochlear implant surgery: A double blinded placebo-controlled trial. Hear. Res..

[16] Rajan G.P., Kontorinis G., Kuthubutheen J. (2013). The effects of insertion speed on inner ear function during cochlear implantation: A comparison study. Audiol. Neurootol..

